# Artificial Intelligence in Medical Education: Transformative Potential, Current Applications, and Future Implications

**DOI:** 10.2196/77127

**Published:** 2026-02-17

**Authors:** Juan S Izquierdo-Condoy, Marlon Arias-Intriago, Laura Montero Corrales, Esteban Ortiz-Prado

**Affiliations:** 1 One Health Research Group, Universidad de Las Américas, Via Nayon S/N, Quito, 170124, Ecuador, +593 995760693; 2 Escuela de Comunicación, Universidad Latina de Costa Rica, San Jose, Costa Rica

**Keywords:** artificial intelligence, medical education, machine learning, adaptive learning systems, future implications

## Abstract

Artificial intelligence (AI) is increasingly influencing medical education by enabling adaptive learning, AI-assisted assessment, and scalable instructional tools. Natural language processing, machine learning, and generative large language models offer innovative ways to support teaching and learning, yet their integration raises ethical, pedagogical, and infrastructural challenges. This viewpoint article aims to examine the current applications, benefits, and challenges of AI in medical education and propose strategies for responsible and effective integration. AI tools such as chatbots, virtual patients, and intelligent tutoring systems enhance personalized and immersive learning. Automated grading and predictive analytics support efficient evaluations, while AI-assisted writing tools streamline content creation. Despite these advances, concerns persist around data privacy, algorithmic bias, unequal access, and diminished critical thinking. Key solutions include AI literacy training, data oversight, equitable infrastructure, and curriculum reform. The FACETS framework offers 6 dimensions (ie, form, application, context, instructional mode, technology, and the SAMR [substitution, augmentation, modification, redefinition model]) to evaluate AI integration effectively. AI offers substantial opportunities to transform medical education, but its adoption must be ethical, equitable, and pedagogically grounded. Strategic frameworks such as FACETS, combined with institutional governance and cross-sector collaboration, are essential to guide implementation so that AI enhances learning outcomes while preserving the humanistic foundations of medical practice.

## Introduction

Artificial intelligence (AI) emerged in the mid-20th century, particularly after the Dartmouth Conference in 1956, as an interdisciplinary field integrating computer science, mathematics, logic, and cognitive science. The first published study on AI in medical education dates back to 1992, although AI has existed much longer than this. Its goal was to simulate human cognitive processes and endow computer systems with human-like abilities, such as reasoning, learning, and decision-making [1]. In recent decades, AI applications have expanded significantly, finding roles in fields such as programming, statistics, preschool education, and university disciplines such as medicine and medical education. However, it was not until the launch of ChatGPT (OpenAI) in 2022 that AI became exponentially popularized, taking on a prominent role in many domains of knowledge. This growth has been driven by technological advances and increasing expectations fueled by globalization and the vast flow of information in today’s interconnected world [2,3].

In the field of sciences, including medicine, biomedicine, and related disciplines, the use of AI extends far beyond the traditional teaching-learning process. It now plays a fundamental role in the entire professional life cycle of medical practitioners. This includes preparation, continuous education, and the acquisition of knowledge. The rapid growth of scientific literature and the constant influx of new information push physicians to seek knowledge from diverse sources, aiming to stay as current as possible. These sources go beyond traditional books or scientific articles, incorporating videos, interactive platforms, virtual classes, and other innovative tools that facilitate learning and professional development [1,2].

AI supports both learning and teaching, leveraging technologies such as natural language processing (NLP), machine learning (ML), and generative pretrained transformer architectures. These technologies enable the creation of innovative educational strategies, revolutionary teaching methods, curriculum design, content development, and the evaluation of various academic processes. This makes AI an indispensable tool for medical education, facilitating both the transmission of knowledge and the continuous improvement of educational systems [4].

The dynamics have changed so rapidly that teachers, educators, students, and trainees are using AI, yet very few have been adequately trained for this purpose. Scientific literature is growing at an unprecedented pace, and while the integration of AI into medical education offers significant benefits, it also presents substantial challenges. One of the most profound critiques involves ethical considerations, the risk of undermining academic integrity, and concerns about students’ reliance on AI for assignments, raising critical questions among educators. As Pineda-de-Alcázar [5] suggests, these technological advances raise fundamental questions about the nature of communication between humans and machines, as AI moves closer to interacting with us in ways that mimic human thought and emotional complexity. Within this context, we explore the applicability and evidence for using AI in biomedical teaching practices.

Against this backdrop, this manuscript addresses the following research question: How are distinct AI modalities being deployed in medical education across learning, assessment, administration, and academic content creation, and what ethically grounded strategies best support their responsible integration? Accordingly, our objective is to examine the applications, benefits, and challenges of AI in medical education across these domains and propose strategies for responsible and effective integration.

## Current Applications of AI in Medical Education

### Overview

AI is rapidly transforming medical education beyond the use of conversational agents such as ChatGPT. Its current impact can be categorized into four main areas: interactive learning tools, intelligent assessment systems, administrative and logistical support, and content creation.

### Interactive Learning Tools

AI-driven virtual assistants and chatbots—such as ChatGPT—facilitate dialogue-based learning, allowing students to practice history taking and enhance communication skills through simulated patient encounters [6]. Moreover, immersive technologies, including virtual reality, intelligent tutoring systems (ITS), medical robotics, and augmented reality, provide hands-on training experiences. These tools promote the development of clinical reasoning, surgical technique, and medical imaging interpretation while delivering personalized feedback and adaptive learning pathways [7,8].

### Intelligent Assessment Systems

AI enhances evaluation processes by enabling automated grading and reducing evaluator bias. NLP can analyze narrative feedback to identify patterns related to competency, professionalism, and potential learner risk [9]. Innovations such as virtual Objective Structured Clinical Examinations (OSCEs) and automated assessments of clinical case presentations streamline performance evaluations [10-13]. In addition, ML contributes to the creation and validation of examination items, although human oversight remains essential to maintain content integrity [14,15].

### Administrative and Logistical Support

In educational administration settings, AI automates the documentation of clinical experiences. For example, NLP applications can map trainee documentation to core competencies with high accuracy (92%‐97%), significantly reducing clerical workload [16-19]. Furthermore, predictive modeling aids in optimizing residency selection and procedural tracking, enhancing the efficiency of academic programs [20,21].

### Content Creation and Academic Writing

AI also supports academic writing by assisting in the drafting of structured clinical notes, research manuscripts, and literature summaries. These tools enhance clarity, reduce writing time, and facilitate effective scholarly communication [22,23] (Table 1).

**Table 1.** Synthesis of artificial intelligence (AI) applications in medical education.

Category and AI application	Description
Virtual assistants and chatbots	
Interactive dialogue systems	Chatbots enable dialogue-based learning, supporting communication, and history-taking practice.
Generative conversational AI	Tools such as ChatGPT simulate realistic patient interactions to develop clinical communication skills.
Simulated medical cases	NLP^a^-driven simulations help assess and improve students’ clinical reasoning and decision-making.
Learning tools, simulation, and VR^b^	
Virtual surgical assistants	AI evaluates surgical performance in simulations, offering feedback for skill refinement.
Virtual patient avatars	2D/3D avatars simulate clinical scenarios to train students in consultations and emergency care.
Virtual patient simulators and ITS^c^	Adaptive platforms offer real-time feedback to strengthen reasoning and procedural skills.
Medical robots	Used for pharmacologic simulations and clinical knowledge assessments in competency-based training.
Robot-assisted surgical training	Enables safe, simulated practice of complex surgical procedures.
Augmented reality in simulations	Augmented reality tools provide interactive training for interpreting imaging (eg, x-rays, CT^d^ scans).
AI-integrated electronic learning platforms	Combine AI tutoring with online and in-person education for personalized learning paths.
NLP for literature processing	NLP tools assist in summarizing and interpreting large volumes of scientific literature.
Intelligent assessment systems	
Unbiased candidate selection	AI reduces demographic bias in residency selection processes.
Test creation	AI generates multiple-choice questions and clinical scenarios for examinations and curriculum.
Automated grading	Systems provide instant feedback on assessments while requiring human oversight.
Content creation and writing support	
AI-assisted academic writing	Tools assist in drafting structured notes and summaries to enhance academic communication.
AI in scientific research	Supports manuscript drafting and editing, with attention to avoiding fabricated content.

a NLP: natural language processing.

b VR: virtual reality.

c ITS: intelligent tutoring system.

d CT: computed tomography.

## Benefits of AI in Medical Education

AI technologies are reshaping medical education by enabling personalized learning, enhancing clinical training, and supporting academic content development. While these advancements offer considerable promise, they also raise significant risks and ethical challenges. Responsible integration of AI requires careful attention to its implications, along with institutional commitment to adapting curricula and policies.

AI-driven technologies provide highly personalized learning experiences that can improve academic performance and clinical competence. One major advantage is the ability to tailor educational content and assessments to the individual learner’s needs, fostering more efficient knowledge acquisition and deeper conceptual understanding [24]. Furthermore, AI-powered simulations—including virtual surgical assistants, patient avatars, and procedural simulators—offer safe, immersive environments where students can refine their diagnostic and technical skills without risk to patients [25].

Exposure to AI also prepares students for its growing role in clinical practice. Familiarity with decision support tools enhances future physicians’ readiness to integrate AI into diagnosis, treatment planning, and patient care [26]. From an instructional perspective, generative AI accelerates the development of educational content by facilitating the creation of multiple-choice questions, clinical vignettes, and tailored teaching modules [23,27,28]. Notably, AI-generated resources have the potential to reduce educational disparities by enhancing access to high-quality materials in underserved regions [23,27].

## Risks and Ethical Considerations

### Risks and Concerns

Despite its benefits, the incorporation of AI into medical training poses several concerns. One is the potential impact on career decisions. Some students may be deterred from entering fields such as radiology due to perceived reductions in future demand, as AI systems become increasingly proficient at image interpretation [29]. Another concern is the limited integration of AI-related content in existing medical curricula, leaving students unprepared to engage with AI-driven health care systems [30]. Additionally, there is the risk of overreliance on AI tools, which may undermine the development of critical hands-on clinical skills, diagnostic reasoning, and human-centered decision-making [31].

### Ethical Considerations

The ethical use of AI in medical education is a growing focus of scholarly and institutional discourse. A key consideration is the preservation of human judgment. While AI can support decision-making, it must not displace the clinician’s responsibility for critical thinking, empathy, and ethical deliberation [32]. Moreover, transparency and accountability are essential. Medical students must be taught to recognize biases, uphold data privacy, and ensure fairness in AI-mediated outcomes. Equitable access is also critical; AI integration must address the digital divide to ensure that learners across different regions and socioeconomic backgrounds benefit equally [29,33,34].

In medical education, AI use may compromise several ethical principles: academic integrity (fabrication or inaccuracy of references), justice (potential biases), nonmaleficence (overreliance on algorithmic outputs by students), and confidentiality (risks of training data extraction). These concerns are supported by empirical findings. For example, in a set of 115 references generated with ChatGPT-3.5 (OpenAI), 47% (n/N) were fabricated, 46% (n/N) inaccurate, and 7% (n/N) authentic and correct [35]; in another analysis of 636 citations, 55% (n/N) were fabricated with GPT-3.5 and 18% with GPT-4 (OpenAI), alongside errors in “real” citations [36]. Editorial practice has likewise documented high similarity indices with plagiarism risk and failures to detect misuse in medical-scientific articles—including AI-generated images with biological errors and the publication of stock large language model (LLM) phrases (eg, “I do not have access to real-time information...”) in papers that passed peer review [37]. Beyond GenAI, computer vision used for instructional purposes (eg, medical imaging or intraoperative video) exhibits performance degradation due to dataset shift, dependence on spurious signals across hospitals or equipment, and vulnerability to adversarial attacks, necessitating external validation, audits, and technical safeguards when incorporated into educational activities [38,39]. From a privacy standpoint, LLMs have demonstrated memorization of training data and the potential for verbatim disclosure, reinforcing the need to avoid uploading protected health information (PHI) and to adopt institutional controls (data policies, privacy-preserving environments, and prior review before use in classrooms or OSCEs) [40,41].

In alignment with the United Nations Educational, Scientific and Cultural Organization (UNESCO) Recommendation on the Ethics of Artificial Intelligence (2021) and the World Health Organization Guidance on Ethics and Governance of AI for Health (2021), we translate principles into practice through five tightly scoped, actionable axes [42,43]: (1) governance and accountability: establish an oversight committee, maintain a public registry of AI systems, and require predeployment Algorithmic Impact Assessments and Data Protection Impact Assessments; (2) human oversight in high-stakes uses: mandate dual human+AI scoring, structured debriefs, and explicit override criteria grounded in clinical or educational judgment; (3) transparency: disclose in syllabi which tools are used and for what purposes and limits, provide model or data cards, and require student disclosure of AI assistance; (4) privacy-preserving data governance: prohibit uploading PHI to public models; enforce data minimization and deidentification, role-based access controls, secure logging, and retention limits; and prefer on-prem and virtual private cloud solutions when processing student data; and (5) equity, safety, and robustness: conduct subgroup bias testing and disparate impact monitoring, pursue cross-site validation where feasible, and set predefined error thresholds with rollback plans. To operationalize these pillars, we propose a staged pathway that begins with pilots in controlled sandbox environments with Algorithmic Impact Assessments or Data Protection Impact Assessments and a clear evaluation plan, then scales gradually with bias and robustness audits and cross-site performance monitoring, and culminates in institutional integration with periodic audits, update protocols, and public reporting. Together, these steps aim to improve learning while safeguarding autonomy, justice, and trust.

## Curriculum and Teaching Strategies for AI

### AI in Curricular Development

To ensure responsible and effective use of AI, medical education should incorporate foundational and applied competencies. Core knowledge areas should include data science, ML, algorithm design, statistics, and basic coding principles [44,45].

Curricular modules should also address clinical applications of AI in domains such as surgical training, radiology interpretation, ophthalmologic analysis, and hematologic diagnostics [46-49]. Ethical and legal education remains vital, with an emphasis on transparency, privacy, and professional accountability [34,50]. Notably, one publication has even proposed establishing a dedicated specialty in Medical Data Sciences to address the growing demand for AI expertise in clinical settings [51].

### Teaching Strategies

Educators are actively exploring pedagogical strategies to integrate AI into undergraduate medical training. AI-enhanced curriculum design, such as the use of chatbots and generative platforms, enables the creation of dynamic learning experiences tailored to student progression and clinical reasoning levels [28]. Similarly, adaptive assessment systems powered by AI can identify individual student weaknesses and provide targeted feedback, thereby optimizing skill development and educational outcomes [23,27].

## Implementation Path to AI in Medical Education: Adoption Trajectory, Challenges, and Solutions

The integration of AI into medical education can be meaningfully interpreted through Oberg’s theory of culture shock, which comprises four progressive phases: honeymoon, frustration, adaptation, and acceptance [52]. This framework provides a valuable lens to examine the emotional, cognitive, and institutional transitions associated with AI adoption in medical education.

### Adoption Trajectory

#### The Honeymoon Phase: Enthusiasm and Idealism

The initial response to AI is often marked by enthusiasm and idealism. During this phase, perspective articles and expert commentaries emphasize AI’s transformative potential, exploring novel applications and offering conceptual frameworks for implementation. However, these early contributions frequently understate practical limitations, ethical dilemmas, and the systemic challenges associated with integration [52].

#### The Frustration Phase: Uncertainty and Resistance

As implementation progresses, institutions often encounter a phase of skepticism and resistance. Concerns may stem from limited technical knowledge, fears of professional displacement, or mistrust in AI’s reliability. Faculty unfamiliar with digital tools may experience anxiety or a sense of obsolescence. Despite its discomfort, this phase is critical, as it catalyzes essential discussions on ethics, equity, and the risks of uncritical adoption [52].

#### The Adaptation Phase: Pragmatic Implementation

Over time, a more balanced perspective emerges, with institutions adopting AI tools in measured and context-sensitive ways. In this phase, AI is viewed not as a panacea but as a complement to existing pedagogical practices. Implementation strategies begin to emphasize design thinking, iterative refinement, and context-specific use cases. Nonetheless, many approaches remain limited in scope and lack a longitudinal vision [52].

#### The Acceptance Phase: Thoughtful Integration and Leadership

In the final phase, AI becomes a normalized and integrated component of the educational ecosystem. Educators demonstrate fluency in AI applications and embed them into curricula, assessments, and research activities with ethical awareness and pedagogical intent. Institutions at this stage offer scalable models characterized by innovation, critical engagement, and a commitment to preserving the humanistic core of medical education [52].

### Persistent Challenges in AI Integration

Despite increasing interest and promising use cases, several challenges continue to hinder the effective integration of AI in medical education. Ethical and legal concerns include risks to data privacy, opaque accountability for AI-supported decisions, and the potential erosion of humanistic principles in teaching and care; AI use must remain aligned with the ethical foundations of medical practice and must respect the clinician-patient relationship [53]. Unreliable or low-quality outputs from generative models, such as ChatGPT and similar tools, may perpetuate outdated, biased, or inaccurate information when these systems are trained on flawed data; inaccuracies in AI-generated educational content can compromise learner safety and clinical competence, underscoring the need for rigorous data curation and active human oversight [1,54]. Scalability and equity barriers arise from limited digital infrastructure and high implementation costs, particularly in low- and middle-income countries, where disparities restrict equitable access to AI-driven tools and may reinforce existing educational inequities [54]. Finally, curricular displacement is a concern: overdependence on AI may inadvertently marginalize foundational clinical reasoning, communication, and hands-on skills if these technologies are introduced without explicit pedagogical safeguards and thoughtful alignment with existing curricula [51].

### Proposed Solutions for Effective and Ethical AI Adoption

To harness the benefits while mitigating potential risks, a comprehensive strategy is essential—one that encompasses ethical considerations, pedagogical advancements, and technological inclusivity. Several complementary approaches can facilitate the responsible adoption of AI in medical education. Strengthening ethical frameworks is critical to ensure transparency, fairness, and respect for patient-centered values throughout AI development and deployment [53]. Enhancing data quality and human oversight is equally important: training AI systems on peer-reviewed data and subjecting outputs to expert review can reduce misinformation and support safe implementation [1,54]. Promoting equitable access involves designing adaptable, low-cost solutions tailored to diverse educational contexts and resource environments, particularly in underserved regions [54]. Reforming curricula to include AI literacy prepares future physicians to engage critically with algorithmic tools, with core competencies that encompass data science, algorithmic reasoning, ethical discernment, and digital fluency [51]. Supporting longitudinal research on the long-term educational and clinical impacts of AI integration will provide the evidence base needed for informed decision-making and continuous improvement [54].

These strategies are further detailed in Table 2, which aligns specific challenges with corresponding solutions to guide effective AI integration in medical education.

**Table 2.** Strategic challenges and solutions for the integration of artificial intelligence (AI) in medical education.

Challenge	Proposed solution
Ethical and legal risks	Strengthen ethical oversight and align AI use with core humanistic values [53]
Unreliable AI-generated content	Ensure high-quality data sources and implement human oversight [54]
Limited scalability and access	Develop accessible, adaptable tools for global use [54]
Curriculum imbalance	Integrate AI literacy into core training without displacing clinical fundamentals [45,51]
Need for evidence-based integration	Support rigorous research on educational impact and long-term outcomes [54]

## Future Directions and Strategic Frameworks for AI in Medical Education

### FACETS as a Unifying Lens for Implementation

**Figure 1.**
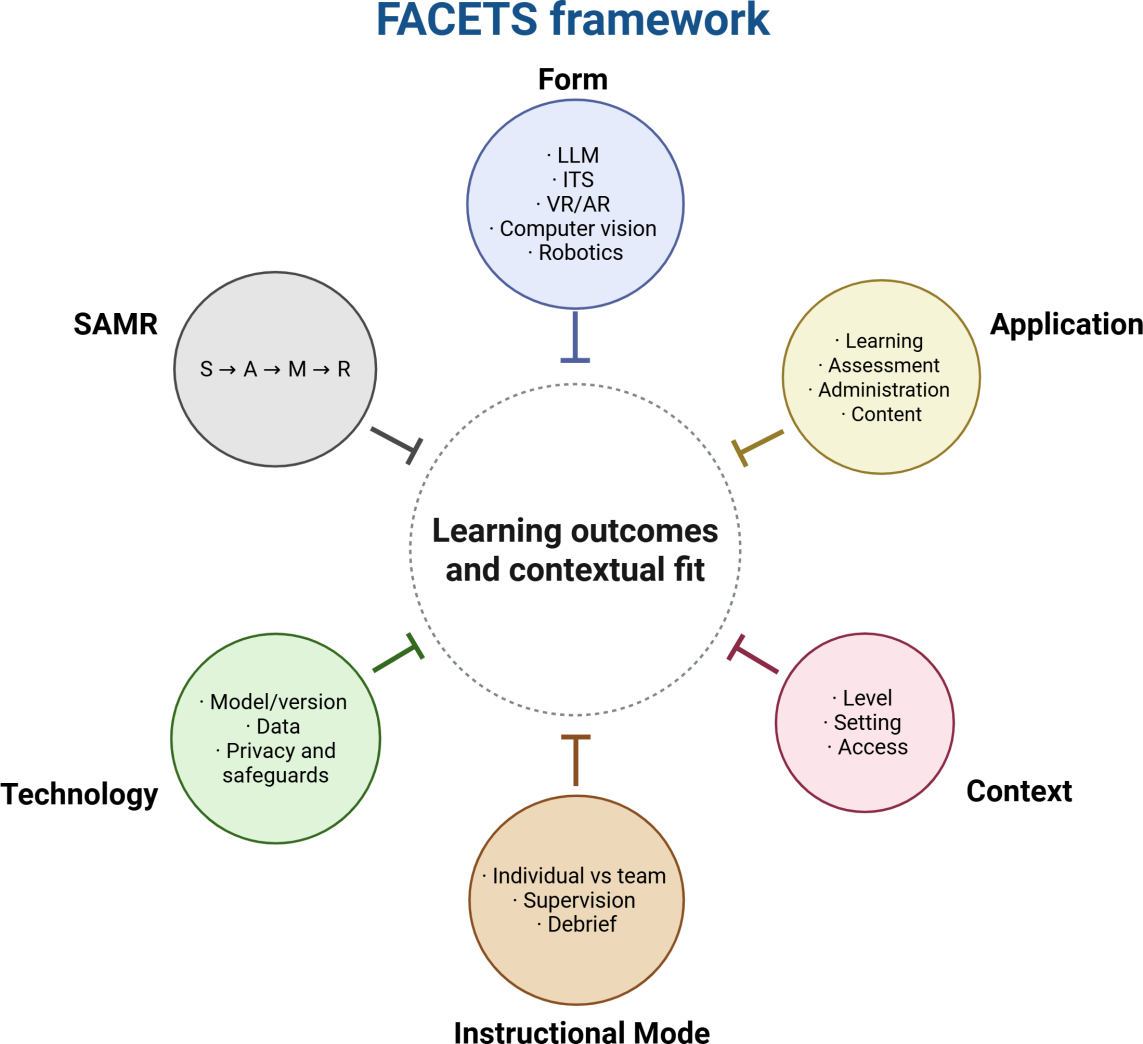
FACETS framework for integrating AI into medical education. This schematic depicts the FACETS framework with six dimensions—form, application, context, instructional mode, technology, and SAMR—arrayed around a central objective: aligning interventions with learning outcomes and contextual fit for the integration of AI in medical education. AI: artificial intelligence; AR: augmented reality; CV: computer vision; GPT: generative pretrained transformer; HITL: human-in-the-loop; ITS: intelligent tutoring system; LLM: large language model; ML: machine learning; NLP: natural language processing; OSCE: Objective Structured Clinical Examination; PHI: protected health information; SAMR: substitution, augmentation, modification, redefinition; VR: virtual reality.

In the current context, recent studies typically address isolated components of AI in medical education, such as specific teaching technologies or environments. However, to advance this field, a more cohesive framework is required that facilitates replication, innovation, and a comprehensive understanding of the educational role of AI [2]. To this end, the FACETS framework has been proposed as a guiding model for future research and implementation in medical education settings. By examining six key dimensions—form, application, context, instructional mode, technology, and the SAMR (substitution, augmentation, modification, redefinition) model—this framework provides a structured approach for assessing how AI tools align with educational objectives (Figure 1). This multifaceted analysis ensures that AI implementations are pedagogically sound and contextually appropriate [2].

The tangible applicability of FACETS can be appreciated by retrospectively mapping published interventions (Table 3). Holderried et al [55] evaluated a GPT-4–based simulated patient that provided immediate, structured feedback for history taking among third-year medical students; they observed more than 99% medically plausible exchanges and near-perfect agreement with a human rater (κ=0.832), although 8 of 45 feedback categories showed lower concordance, underscoring the need for human oversight. Similarly, Luordo et al [56] used GPT-4 to grade OSCE reports from 96 students, finding high concordance with human graders (intraclass correlation coefficient=0.77 for single measures; 0.91 for average measures), systematically stricter AI scoring (AI mean 8.88, SD 2.96 vs experts’ mean 12.39, SD 3.22; mean difference −3.51 points), and substantially shorter grading times (~24 min vs 2‐4 h). Taken together, although the original studies did not explicitly use FACETS, this mapping supports the utility of the FACETS framework as an analytic lens to describe, compare, and guide future implementations of AI in medical education, particularly in undergraduate contexts.

Beyond the specific cases mapped earlier, FACETS can also be used to anticipate and assess emerging applications of AI in medical education—ranging from ITS and personalized learning platforms to chatbots (eg, ChatGPT) and intraoperative video analysis—by aligning each use case with pedagogical intent, context, and level of transformation [2].

**Table 3.** Form, application, context, instructional mode, technology, SAMR^c^ (FACETS) mapping of 2 real-world artificial intelligence (AI) interventions in medical education.

Dimension (FACETS)	Case 1—prospective study; 106 conversations (1894 question-answer pairs) [55]	Case 2—observational study; 96 students; AI versus human graders [56]
Form	LLM^a^ chatbot simulating a patient with structured feedback	Automated scoring system for OSCE^b^ reports (LLM)
Application	History-taking practice+evaluation of coverage of critical items	Formative or summative grading of reports using an institutional checklist
Context	Third-year students, European medical school, individual practice	Hospital teaching unit; 96 students in a real OSCE
Instructional mode	AI-guided practice with comparison against a human rater (for debrief)	AI-assisted assessment with expert benchmarking (human-in-the-loop)
Technology	GPT-4; analysis of response plausibility and category-based feedback quality	Batch GPT-4 pipeline; comparison with human graders; time logging
SAMR (level of transformation)	Augmentation → modification (replaces SP+adds rubric-aligned feedback or analytics)	Augmentation → modification (from manual scoring to automated with traceability or speed)

a LLM: large language model.

b OSCE: objective structured clinical examination.

c SAMR: substitution, augmentation, modification, redefinition.

### Emerging Applications and Integrative Synthesis for Decision-Making

As AI becomes more deeply embedded in health care delivery, its incorporation into medical education continues to represent a critical yet uneven frontier. Although AI tools have shown promise in diagnosis, therapeutic planning, and operational efficiency, educational adoption has lagged—both in scale and in the rigor of implementation and evaluation. Key barriers include heterogeneous technological infrastructure—particularly in resource-limited settings—and limited AI literacy among students and faculty [57,58]. These challenges are compounded by the fact that many medical students, despite their interest in AI’s potential, express anxiety about downstream labor market implications [59,60].

Beyond the case-based exemplars mapped with FACETS, diverse emerging applications in medical education share several salient features: they prioritize deliberate practice with adaptive feedback and competency-aligned performance traceability; they operate predominantly at the augmentation and modification levels of the SAMR model (with few achieving true redefinition); and they rely on high-quality data (interaction logs, rubrics, audio-video) that demand psychometric validation, bias and drift monitoring, and robust privacy safeguards. The evidentiary base remains heterogeneous, with single-site pilots and intermediate metrics predominating over longitudinal outcomes. The human factor remains essential—explicit instructional design, structured debriefing, and human-in-the-loop approaches are needed to calibrate criteria and prevent overreliance. Absent targeted faculty development and institutional processes (including data governance, audits, and reporting standards), these solutions risk amplifying equity gaps.

To operationalize these principles and translate them into programmatic, monitorable curricular decisions, it is helpful to note that these common features are especially evident in intelligent tutoring systems (ITSs), clinical chatbots, AI-assisted assessment, personalized learning platforms, virtual reality and AI simulators, anatomy tools, and intraoperative video analytics (Table 4). Building on that foundation, cross-cutting applications that enable and govern implementation across workstreams assume particular importance: (1) multimodal adaptive analytics, which fuse text, audio-video, and interaction traces to personalize pacing and content while flagging early risk at the learner or course level [61]; (2) learning analytics dashboards, which integrate competencies, assessment evidence, and progression signals to guide just-in-time feedback, remediation, and coaching for students and instructors [62]; and (3) educational clinical decision support, implemented in deidentified, guideline-constrained sandbox environments to teach evidence retrieval, uncertainty appraisal, and human-AI teaming in diagnostic and therapeutic planning [63,64] (Table 4).

Effective adoption of these cross-cutting layers requires the very institutional capacities and safeguards they presuppose. Curricula must evolve to incorporate AI training that fosters both technical competence and ethical responsibility [2]. This entails not only understanding AI tools but also recognizing their limitations, biases, and societal implications. Cross-sector collaboration among educational institutions, technology developers, and researchers is crucial to establishing universal digital literacy standards that equip learners and faculty to critically evaluate AI-generated content and apply such tools responsibly in clinical and educational settings [4].

**Table 4.** Emerging artificial intelligence (AI) applications in medical education.

Application	Description
Intelligent tutoring systems	AI-powered platforms enhancing decision-making and clinical reasoning [65]
AI-assisted learner assessment	Automated grading using NLP^a^ and semantic analysis for case summaries [66]
Chatbots (eg, ChatGPT)	Teaching clinical management, USMLE^b^ prep, communication skills, USMLE prep, communication skills [67,68]
Personalized learning platforms	Tailored learning paths and feedback for individualized student development [69]
Surgical simulations (VR^c^/AI)	Virtual reality tools for surgical skill training and evaluation [70]
Enhanced anatomy education	AI-assisted tools for deeper learning and retention in anatomy [71]
AI in admissions	Tools supporting application reviews and personal statement development [72]
AI-generated art	Improving patient education through visual storytelling [73]
Intraoperative video analysis	Teaching competency-based assessments via machine learning [74]
Multimodal adaptive analytics	Fuses text, audio-video, interaction data to personalize pacing and flag early risk [61]
Learning analytics dashboards	Aggregates competencies and assessment traces for just-in-time feedback and monitoring [62]
AI-supported CDS^d^ for education	Guideline-constrained sandbox CDS to teach evidence retrieval, uncertainty, and human-AI teaming [63]

a NLP: natural language processing.

b USMLE: United States Medical Licensing Examination.

c VR: virtual reality.

d CDS: clinical decision support.

AI-powered platforms are expanding personalized learning by tailoring educational content to individual needs. Technologies such as virtual patients, augmented reality simulations, and mobile platforms offer dynamic, interactive experiences that increase engagement and broaden access to high-quality education, particularly in resource-limited regions. Nonetheless, the risk of depersonalizing medical practice—together with substantial concerns about data privacy—underscores the need for rigorous ethical and regulatory frameworks that engage all stakeholders in the educational process [75].

Given AI’s expanding role in health care, prohibiting its use in education is neither practical nor beneficial. Institutions should instead establish comprehensive guidelines to ensure that AI-generated content remains reliable, relevant, and ethically sound. A structured, responsible approach—grounded in robust educational infrastructure, interdisciplinary collaboration, and consistent regulatory oversight—has the potential to improve learning outcomes and, ultimately, strengthen patient care.

**Figure 2.**
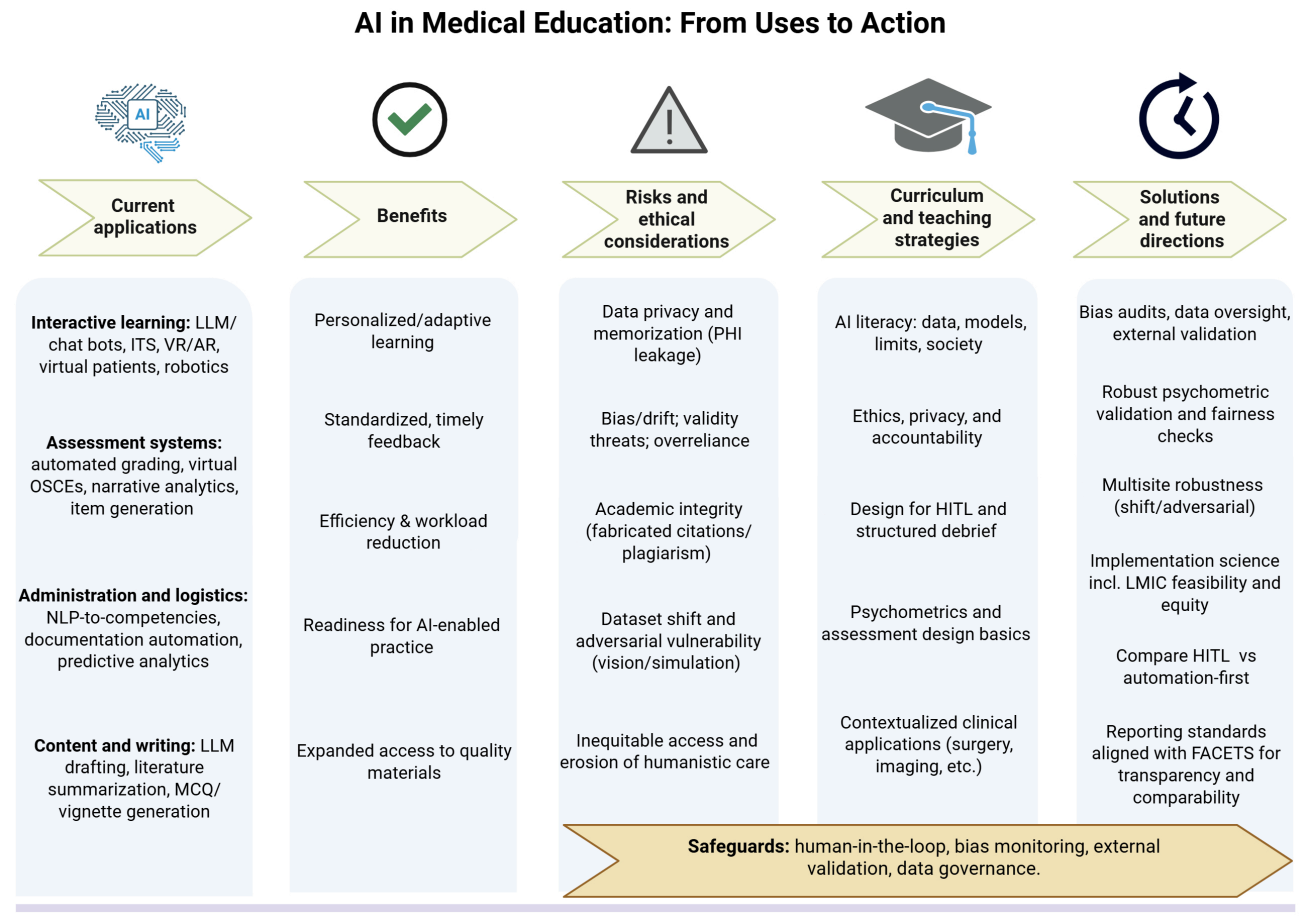
Schematic overview of AI applications in medical education. This figure synthesizes a practical pathway for AI uses in medical education. From left to right, it maps current applications—interactive learning, assessment systems, administration or logistics, and content or writing—to the benefits they enable; the risks and ethical considerations they entail; and the curriculum and teaching strategies required for responsible use. The rightmost column summarizes solutions and future directions: bias audits and data oversight; robust psychometrics and fairness checks; multisite robustness testing; implementation science (including LMIC feasibility and equity); comparative studies of HITL versus automation-first approaches; and reporting standards aligned with FACETS to support transparency and comparability. The bottom banner highlights cross-cutting safeguards—human-in-the-loop processes, bias monitoring, external validation, and data governance—that should accompany all deployments. AI: artificial intelligence; AR: augmented reality; FACETS: form, application, context, instructional mode, technology, and SAMR (substitution-augmentation-modification-redefinition); HITL: human-in-the-loop; ITS: intelligent tutoring system; LLM: large language model; LMIC: low- and middle-income country; MCQ: multiple-choice question; NLP: natural language processing; OSCE: Objective Structured Clinical Examination; PHI: protected health information; VR: virtual reality.

As a synthesis of the manuscript’s arguments, we recommend a concise, actionable set of practices: (1) report every AI pilot with a FACETS-aligned template to ensure transparency and comparability; (2) maintain human oversight for high-impact uses (eg, dual scoring with override), reserving automation-first approaches for low-risk contexts with active monitoring; (3) conduct predeployment audits for bias, drift, and adversarial robustness and, where appropriate, cross-site validation before scaling; (4) operate privacy-preserving workflows (no PHI in public LLMs, institutional sandboxes, data minimization or deidentification, role-based access, secure logging and retention); (5) deploy learning analytics dashboards tied to competency frameworks with ongoing psychometric monitoring; and (6) evaluate equity and feasibility using implementation science designs, including in resource-limited settings with disparate impact monitoring. This roadmap is summarized in Figure 2.

## Conclusions

AI is redefining the landscape of medical education by offering innovative tools that enhance learning, assessment, and administrative processes. AI-driven platforms, such as ITS, virtual simulations, and generative models, provide personalized and interactive educational experiences, improving knowledge acquisition and clinical skills while streamlining administrative tasks to increase efficiency and reduce educator workload. These advancements hold immense potential to revolutionize medical education further. By fostering interdisciplinary collaboration, investing in robust infrastructure, and prioritizing ethical considerations, stakeholders can harness AI’s capabilities to enhance learning outcomes and ultimately improve patient care.

At the same time, integrating AI into medical education presents several challenges. Ethical concerns such as data privacy, algorithmic bias, and the potential erosion of humanistic care must be carefully addressed. Technological disparities, particularly in low-resource settings, also hinder equitable AI adoption. To meet these challenges, medical curricula must evolve to include AI training that promotes both technical competence and ethical awareness, encompassing knowledge of AI tools, their limitations, and their societal implications. Cross-sector collaboration among educational institutions, technology developers, and researchers is essential to establishing universal digital literacy standards. These standards should equip students and educators to critically assess AI-generated content and use these tools responsibly in both educational and clinical settings.

Looking ahead, priorities include longitudinal studies linking AI-enabled instruction to validated learning outcomes and real-world performance; rigorous psychometric validation of AI-assisted assessments with routine bias audits; multisite robustness testing—addressing dataset shift and adversarial risk—especially for vision and simulation; implementation science work in diverse, including low-resource, settings on feasibility, cost-effectiveness, and equity; comparative evaluations of human-in-the-loop versus automation-first designs and their impact on critical thinking and professional identity; and FACETS-aligned reporting standards to strengthen transparency, reproducibility, and cross-study comparability. With ethically grounded design and cross-sector collaboration, AI can enhance learning while preserving the humanistic foundations of medical practice.

## Abbreviations

AI: artificial intelligence
FACETS: form, application, context, instructional mode, technology, SAMR (framework components)
ITS: intelligent tutoring system
LLM: large language model
ML: machine learning
NLP: natural language processing
OSCE: Objective Structured Clinical Examination
PHI: protected health information
SAMR: substitution, augmentation, modification, redefinition
